# DNA Methylation Manipulation of Memory Genes Is Involved in Sevoflurane Induced Cognitive Impairments in Aged Rats

**DOI:** 10.3389/fnagi.2020.00211

**Published:** 2020-08-18

**Authors:** Cheng Ni, Min Qian, Jiao Geng, Yinyin Qu, Yi Tian, Ning Yang, Shuai Li, Hui Zheng

**Affiliations:** ^1^Department of Anesthesiology, National Cancer Center/National Clinical Research Center for Cancer/Cancer Hospital, Chinese Academy of Medical Sciences and Peking Union Medical College, Beijing, China; ^2^Department of Anesthesiology, Peking University Third Hospital, Beijing, China

**Keywords:** DNA methylation, epigenetic, anesthesia, memory gene, cognitive impairment

## Abstract

DNA methylation is an essential epigenetic mechanism involving in gene transcription modulation. An age-related increase in promoter methylation has been observed for neuronal activity and memory genes, and participates in neurological disorders. However, the position and precise mechanism of DNA methylation for memory gene modulation in anesthesia related cognitive impairment remained to be determined. Here, we studied the effects of sevoflurane anesthesia on the transcription of memory genes in the aged rat hippocampus. Then, we investigated changes in DNA methylation of involved genes and verified whether dysregulated DNA methylation would contribute to anesthesia induced cognitive impairment. The results indicated that sevoflurane anesthesia down-regulated the mRNA and protein levels of three memory genes, *Arc*, *Bdnf*, and *Reln*, which were accompanied with promoter hypermethylation and increased *Dnmt1*, *Dnmt3a*, and *Mecp2* expression, and finally impaired hippocampus dependent memory. Furthermore, inhibition of DNA hypermethylation by 5-Aza rescued sevoflurane induced memory gene expression decrease and cognitive impairment. These findings provide an epigenetic understanding for the pathophysiology of cognitive impairment induced by general anesthesia in aged brain.

## Introduction

A progressive loss of cognitive function characterized by impairments in memory is common in aging and creates a context favorable for the development of neurodegenerative diseases such as Alzheimer’s disease (AD) (Barter and Foster, 2018). However, the rate and severity of cognitive aging vary widely across the elderly. Recently, epigenetic modification emerges as a crucial mechanism in shaping phenotypic differences of cognitive aging through gene−environment interactions (Barter and Foster, 2018). DNA methylation, one of the most extensively investigated epigenetic mechanisms, is an essential mediator of memory associated gene transcription, typically resulting in transcriptional silencing and loss of gene function. DNA methylation is catalyzed by DNA methyltransferases (DNMTs), including DNMT1, DNMT3a and 3b. These enzymes transfer a methyl group from S-adenosylmethionine (SAM) to a cytosine, and most commonly occurs at cytosines followed by a guanine residue, referred to as CpG sites of DNA promoter regions (Okano et al., 1999). An age-related increase in promoter methylation has been observed for neuronal activity and memory genes and is involved in neurological disorders (Delgado-Morales et al., 2017). Affected genes implicated in learning and memory notably include the brain derived neurotrophic factor (*Bdnf*) (Lubin et al., 2008), activity-regulated cytoskeletal-associated protein (*Arc*) (Epstein and Finkbeiner, 2018), early growth response 1 (*Egr1*, also known as Zif268) (Sun et al., 2019), *Reln* (Stranahan et al., 2013; Pujadas et al., 2014) and *Ppp3ca* (also known as calcineurin) (Miller et al., 2010).

Approximately 30% of the population aged 65 years or more are exposed to some form of anesthesia annually (Evered et al., 2017). Aging induced neural degenerative changes predispose them to a higher incidence of perioperative neurocognitive disorders (PNCD), including postoperative cognitive dysfunction (POCD) (Moller et al., 1998; Monk et al., 2008; Evered et al., 2018). Although the causes of POCD are complicated and remain to be elucidated, inhaled anesthetics are now being recognized as a potentially significant risk to cognitive performance at extreme age (Jevtovic-Todorovic et al., 2013; Schulte et al., 2018). Our studies and others revealed that exposure to inhaled anesthetics could result in synaptic and cognitive dysfunction (Ni et al., 2015, 2017). Recent work has begun to investigate anesthesia triggered epigenetic modifications within the neonatal rats (Chastain-Potts et al., 2019). However, the manipulation of DNA methylation and its precise mechanism for memory genes in the aged hippocampus and their relationships with anesthesia related cognitive variation remained to be determined. In the present study, we tested whether sevoflurane, one of the most commonly used inhaled anesthetics for general anesthesia, would suppress the transcription of memory genes in the hippocampus of aged rats. Then, we investigated changes in the profile of DNA methylation of involved genes and verify whether dysregulated DNA methylation within hippocampus would contribute to anesthesia induced cognitive impairment. We aim to elucidate the epigenetic mechanisms underlying neurocognitive disorders induced by anesthesia in the aged brain.

## Materials and Methods

### Animals and Anesthesia

Male Sprague-Dawley rats, 18-month old, weighing 550–600 g, were used in the studies. Before sevoflurane anesthesia, the rats were maintained on a standard housing condition with food and water *ad libitum* for 2 weeks. The animal experiments were performed in accordance with the guide for the care and use of laboratory animals and the protocol was approved by the local biomedical ethics committee (No. LA2018085).

Minimum alveolar concentration (MAC) of sevoflurane for rats has been reported as 2.4∼2.7% (Li et al., 2014). In our study, rats were randomly assigned to control or sevoflurane group. The rats in anesthesia group received 2.5% sevoflurane in 100% oxygen for 4 h in an anesthetizing chamber, while the control group received 100% oxygen at an identical flow rate for 4 h in an identical chamber. The rats breathed spontaneously, and sevoflurane and oxygen concentrations were monitored continuously (Datex, Tewksbury, MA, United States). The temperature of the anesthetizing chamber was controlled to maintain the rectal temperature of rats at 37 ± 0.5°C. This anesthesia protocol has been shown not to significantly alter values of blood pressure and blood gas in the preliminary studies. Anesthesia was terminated by discontinuing sevoflurane and placing animals in a chamber containing 100% oxygen until 20 minutes after the recovery of consciousness. The animals were then returned to individual home cages until sacrifice. Rats were sacrificed by decapitation 3 h after anesthesia. The brain tissues were removed rapidly, and the hippocampus was dissected out and frozen in liquid nitrogen.

### Cell Culture and Treatments

C6 rat glioma cells were used in the studies. The cells were cultured in F-12K medium (Gibco^TM^, Thermo Fisher Scientific, Waltham, MA, United States) containing 2.5% fetal bovine serum, 15% horse serum, 100 U/ml penicillin, and 100 μg/ml streptomycin. Before treatments, cells were seeded in 6-well plates, with one million cells in 1.5 ml cell culture media per well, as described in our previous studies (Ni et al., 2017). The cells were randomly assigned to a treatment or control group. In treatment group, the cells were treated in a sealed plastic box in a 37°C incubator, with 4.1% sevoflurane, plus 21% O2 and 5% CO2, delivered from an anesthesia machine for 4 h as described previously (Dong et al., 2009). The cells in the control group received vehicle gas in the same condition. A Datex infrared gas analyzer (Puritan-Bennett, Tewksbury, MA, United States) was used to continuously monitor the delivered concentrations of carbon dioxide, oxygen and sevoflurane. Then the treated cells were harvested and frozen in liquid nitrogen.

### DNA Methylation Modulation

To conduct the *in vivo* induction of DNA hypomethylation, rats were randomly assigned to control, 5-Aza-2′-deoxycytidine (5-Aza), sevoflurane or sevoflurane +5-Aza group. 5-Aza (Abcam, Cambridge, United Kingdom) is a DNMT1 activity inhibitor and could induce DNA hypomethylation (Christman, 2002). It was dissolved in sterile water and injected intraperitoneally 30 min before anesthesia with 0.5 mg/kg dosage chosen based on the preliminary study and a previous study (Williams-Karnesky et al., 2013). The control and sevoflurane groups received a vehicle injection (sterile water).

For the *in vitro* induction of DNA hypomethylation, the cells were randomly assigned to control, 5-Aza, sevoflurane or sevoflurane +5-Aza group. 5-Aza was given 60 min before sevoflurane treatment in 5-Aza or sevoflurane +5-Aza group, and the concentration was 10 μM based on a previous study (Tan et al., 2017). For the *in vitro* induction of DNA hypermethylation, the cells were randomly assigned to control, S-(5-Adenosyl)-L-methionine disulfate tosylate (SAM), sevoflurane or sevoflurane + SAM group. SAM (Methyl donor, Abcam, Cambridge, United Kingdom) was also given 60 min before sevoflurane treatment in SAM or sevoflurane + SAM group, and the concentration was 100 μM based on a previous study (Maddocks et al., 2016).

### RNA Extraction and Quantification

Total RNAs were isolated from the hippocampi and cells using TRIzol reagent (Invitrogen, Carlsbad, CA, United States), then were digested with RNase-Free DNase to remove residual DNAs. The RNA concentrations were analyzed using the Nanodrop 2000 (Thermo Fisher Scientific), then total RNA (2 μg) was reverse-transcribed using the GoScriptTM Reverse Transcription System (Promega).

### Quantitative Real-Time PCR (qPCR)

Quantitative real-time PCR was performed on CFX96 Real-Time PCR Detection System (Bio-Rad, Hercules, CA, United States). Amplification mixture consisted of PowerUpTM SYBR^®^ Green master mix (Thermo Fisher Scientific), 10 μM forward and reverse primers (Invitrogen, Carlsbad, CA, United States) and approximately 1.5 μl of cDNA template. Primer sequences were obtained from the literature and checked for their specificity through *in silico* PCR. The forward and reverse primers are shown in Table 1. Amplification was carried out with an initial denaturation step at 95°C for 2 min followed by 45 cycles of 95°C for 10 s, 55°C for 30 s and 60°C for 30 s, then 65°C for 2 min in 10 μl reaction volume. All reactions were run in duplicate and the results were averaged from 6 independent studies. qPCR was quantified in two steps. First, β-actin levels were used to normalize target gene levels (ΔCycle threshold (ΔCt) = Ct_target gene_ − Ct_β–actin_, target gene level = 2−ΔCt). Second, the target gene levels of sevoflurane group were presented as the percentage of those of control group, and 100% of the target gene levels referred to the control levels.

**TABLE 1:** The primer sequences used for qPCR.

Genes	Orientation	Sequence (5′ to 3′)
*Arc*	Forward	CTCCAGGGTCTCCCTAGTCC
	Reverse	TGAGACCAGTTCCACTGCTG
*Bndf*	Forward	GTGACARTATTAGCGAGTGGG
	Reverse	GGGTAGTTCGGCATTGC
*Reln*	Forward	AAACTACAGCGGGTGGAACC
	Reverse	ATTTGAGGCATGACGGACCTATAT
*Egr1*	Forward	ACGCCATATAAGGAGCAGGA
	Reverse	TATTGGAACAGCAGCAGTGG
*Ppp3ca*	Forward	TGAACATAGCCAGGGTCACA
	Reverse	GAAGGCGACGAGTAAACAGC
*Dnmt1*	Forward	AAATCCGTCTGTGGAAGGTG
	Reverse	GCGGTAGAAAAGCCAATGAG
*Dnmt3a*	Forward	TGTGAATGATAAGCTGGAGTTGC
	Reverse	GGTGGTAATGGTCCTCACTTTG
*Dnmt3b*	Forward	GAATTTGAGCAGCCCAGGTTG
	Reverse	AAGAAGAGCCTTCCTGTGCC
*Mecp2*	Forward	GGTAGAAAGCCTGGGAGTGT
	Reverse	TTCTTGATGGGGAGCACAGT
*Actb*	Forward	AGAGCTATGAGCTGCCTGA
	Reverse	AATTGAATGTAGTTTCATGGATG

### Immunofluorescence

Immunofluorescence was performed in order to determine the expression of Arc, BDNF and Reelin in the hippocampus, as described in our previous studies (Ni et al., 2015). The rat hippocampus was fixed with 4% paraformaldehyde for 24 h, cryoprotected with 30% sucrose for 48 h, and sectioned using a cryostat (Cryotome E, Thermo Fisher, MA, United States). Coronal sections (10 μm thickness) were incubated with Arc antibody (1:200 dilution; Abcam, Cambrige, United Kingdom), BDNF antibody (1:1000 dilution; Abcam, Cambrige, United Kingdom) or Reelin antibody (1:500 dilution; Abcam, Cambrige, United Kingdom) overnight at 4°C, followed by incubation with goat anti-rabbit IgG conjugated to Alexa Fluor^®^ 488 (1:400 dilution; Servicebio, Wuhan, China) for 50 min at room temperature. Nuclei were subsequently counterstained with DAPI (1:5000 dilution; Servicebio, Wuhan, China) for 10 min at room temperature. Images were captured using a Nikon Eclipse Ti confocal microscope. Due to the results of previous studies (Lee and Kesner, 2002), the hippocampal subregions CA1 and DG serve important roles in retrieving contextual memory after a long time period (i.e., 24 h), these two regions were analyzed for Arc, BDNF and Reelin expressions. Images were acquired by Nikon Eclipse Ti-S microscope (Nikon Corp., Tokyo, Japan).

### MassARRAY EpiTYPER Assay

DNA methylation was quantified by the mass spectrometry-based method MassARRAY EpiTYPER compact MALDI-TOF (Agena Bioscience Inc., San Diego, CA, United States), and the result data were deposited in Dryad Digital Repository^1^. In brief, DNA was extracted from C6 glioma cells and rat hippocampi using Cell/Tissue DNA Extraction Kit (BioTeke, Beijing, China), then was bisulfite converted using Zymo Research EZ DNA Methylation Kit (Zymo Research, Irvine, CA, United States). Primers were designed using the EpiDesigner Software^2^, and their sequences are listed in Table 2. PCR amplification was carried out in an 8 μl reaction volume containing 0.8 μl of 10 × PCR Buffer, 0.8 μl of dNTPs, 0.1 μl PCR enzyme, 0.2 μl of each primer, 1.0 μl of bisulfite-converted DNA and H_2_O, and the amplification was carried out with an initial denaturation step at 94°C for 4 min followed by 45 cycles of 94°C for 20 s, 56°C for 30 s, and 72°C for 1 min, then final extension at 72°C for 3 min. After PCR and Shrimp Alkaline Phosphatase treatment, fragments were ligated to a T7 promoter segment, and then transcribed into RNA. The synthesized RNA was cleaved with RNase A and all cleavage products were analyzed on a mass spectrometer, according to the manufacture’s protocol. The generated mass signal patterns were translated into quantitative DNA methylation levels of different CpG sites of the selected genes by MassARRAY EpiTYPER Analyzer software. The locations of promoter regions encompassing the transcription start and the number of CpG sites assessed in the promoter regions are listed in Table 2. The results were processed and analyzed by the MassARRAY Workstation software. All measurements were performed in triplicate and the average was used for statistical analysis.

**TABLE 2:** The primer sequences and assessed locations for Bisulfite sequencing PCR.

Genes	Orientation	Sequence (5′ to 3′)	Location	CpG sites
*Arc*	Forward	aggaagagagGGTAGAGGAGAGTGT TTTTGGTTTT	−274 to +318	42
	Reverse	cagtaatacgactcactatagggagaaggctACA CTTACCAATCTACAAAATCACATT		
*Bdnf Primer I*	Forward	aggaagagagATTGTGATTTTTTTGGT AAAAAGGA	−644 to −99	16
	Reverse	cagtaatacgactcactatagggagaaggctCCA AAACCCACCTTCTAAAACTTAT		
*Bdnf Primer II*	Forward	aggaagagagTTATTTTTTAGTATTTG TTGGGGAGA	−634 to −36	6
	Reverse	cagtaatacgactcactatagggagaaggctCCTT TCCATATATAAAAACATTACCCA		
*Bdnf Primer III*	Forward	aggaagagagTGTTTATTTATAATGAA ATGGGTAATGT	−1216 to −730	18
	Reverse	cagtaatacgactcactatagggagaaggctACC AAAAATCTATTCCAACCTACAC		
*Bdnf Primer IV*	Forward	aggaagagagTTGTTGTTGTTTAGATG ATGAAAGG	−245 to +346	18
	Reverse	cagtaatacgactcactatagggagaaggctACC CACCTTTTTCAATCACTACTTA		
*Bdnf Primer VI*	Forward	aggaagagagGAGTTTTGGGGTTAAG TAGTTGGTT	−192 to +349	35
	Reverse	cagtaatacgactcactatagggagaaggctCCT CAAAATCCACACAAAACTCTC		
*Reln*	Forward	aggaagagagGTAGTTAGGTTGAAA GGGAGATTGG	−1383 to −834	17
	Reverse	cagtaatacgactcactatagggagaaggctTAA TACCCTTTTCCCAAACTCAAAC		

### Fear Conditioning Test (FCT)

The FCT (Xeye CPP, Beijing MacroAmbition S&T Development, Beijing, China) was performed as described in previous studies (Dong and Li, 2014; Cheng et al., 2015) with modification. Briefly, 3 h after the sevoflurane anesthesia, rats were placed in the context chamber to acclimate for 180 s. Then they received a 2-Hz pulsating tone (80 dB, 3,600 Hz) for 60 s co-terminated with a mild foot shock (0.8 mA, a 0.5 s). Contextual FC memory was assessed 48 h and then 7 days after conditioning, respectively. A contextual test was performed in the same chamber for but with no cues (tone or shock). A cued test was performed by the presentation of same tone without shock in an alternative context with distinct visual and tactile cues. Freezing behavior, recognized as lack of movement except for respiratory efforts, was recorded for 180 s by video and analyzed using Xeye FCT software. The freezing time was used as an indicator of memory formation during training and memory retrieval after anesthesia. Hippocampal dependent memory was assessed by the freezing time during exposure to a novel context, while hippocampal independent memory was assessed by the freezing time during exposure to the conditional stimulus (tone) (Chowdhury et al., 2005).

### Statistical Analysis

Statistical analysis was performed with Graphpad Prism 7.0 software. Quantitative data were presented as the mean ± SD and tested to be normally distributed. Based on the preliminary data, six rats per group was chosen as the sample size for qPCR and DNA methylation studies while twelve per group was selected for FCT experiment to yield a 90% power and 95% significance. The Shapiro-Wilk normality test has been used to test if the values of each group come from normal distribution. Non-paired two-tailed Student’s *t*-test was used to determine significant differences between two groups. One-way ANOVA was used to analyze significant differences between multiple groups, Bonferroni *post hoc* analyses were conducted if the main effects were significant. Two-way ANOVA was used to assess the interaction of 5-Aza or SAM with sevoflurane and to test the hypothesis of whether sevoflurane could influence memory gene expression through DNA methylation pathway. Bonferroni *post hoc* analyses were conducted if the main effects were significant. *P* < 0.05 was considered statistically significant.

## Results

### Sevoflurane Anesthesia Decreased *Arc*, *Bdnf*, and *Reln* mRNA and Protein Expression Levels in the Hippocampus of Aged Rats and C6 Glioma Cells

**FIGURE 1:**
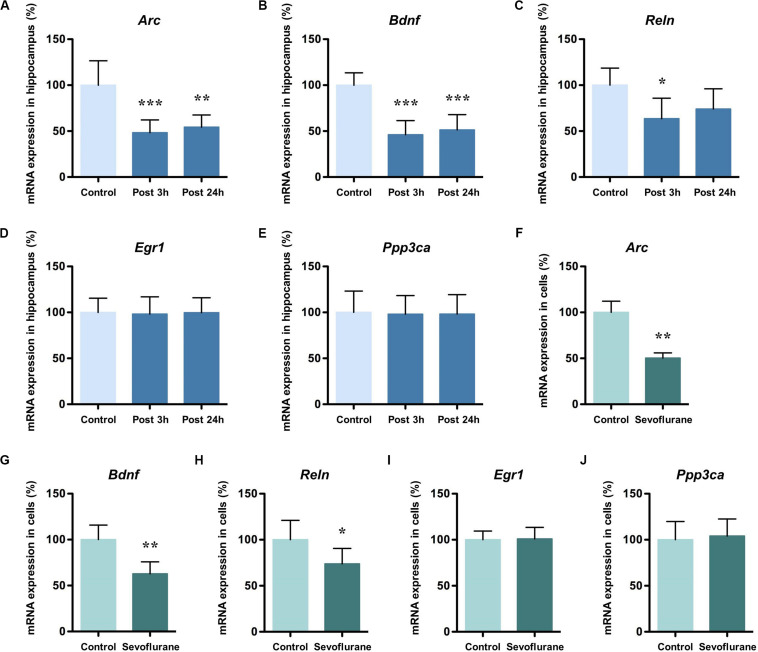
Sevoflurane decreased mRNA levels of *Arc*, *Bdnf* and *Reln* in the hippocampus of aged rats and C6 glioma cells. The mRNA levels of *Arc*, *Bdnf* and *Reln* in the hippocampus of aged rats decreased significantly 3 h afte*r* 4 h 2.5% sevoflurane anesthesia as compared with control condition, and the mRNA levels of *Arc* and *Bdnf*, but not *Reln*, still decreased significantly 24 h after anesthesia **(A–C)**. The mRNA levels of *Egr1* and *Ppp3ca* did not change after anesthesia **(D,E)**. The mRNA levels of *Arc, Bdnf*, and *Reln* in C6 glioma cells decreased significantly after sevoflurane treatment **(F–H)**, but *Egr1* and *Ppp3ca* did not change after treatment **(I,J)**. *n* = 6 in each group of *in vitro* and *in vivo* studies. *P < 0.05, **P < 0.01, and ***P < 0.001 compared with the control group.

To find out the gene markers affected by sevoflurane anesthesia within hippocampus of aged rats, we began by examining the expression of five memory genes *Arc*, *Bdnf*, *Reln*, *Egr1* and *Ppp3ca* (Hendrickx et al., 2014; Sachser et al., 2016; Duclot and Kabbaj, 2017) using quantitative real-time PCR. Compared with control condition, the mRNA levels of *Arc* (48.36 ± 13.82 vs. 100.00 ± 26.58, *P* = 0.0005), *Bdnf* (46.11 ± 15.36 vs. 100.00 ± 13.4, *P* = 0.0001), and *Reln* (63.61 ± 22.24 vs. 100.00 ± 18.60, *P* = 0.0181) decreased significantly 3 h after 2.5% sevoflurane anesthesia for 4 h. The decreased mRNA expression of *Arc* (54.30 ± 13.30, *P* = 0.0015), *Bdnf* (51.26 ± 16.62. *P* = 0.0001) persisted for 24 h after anesthesia, but not for *Reln* (74.00 ± 22.10, *P* = 0.0984, Figures 1A–C). No significant differences were observed in the mRNA levels of *Egr1* and *Ppp3ca* 3 h and 24 h after sevoflurane anesthesia versus control condition (*Egr1*, 98.22 ± 18.71 and 99.70 ± 16.29 vs. 100.00 ± 15.44, *P* = 0.9759 and 0.9993, respectively; *Ppp3ca*, 98.06 ± 20.21 and 98.20 ± 21.12 vs. 100.00 ± 23.22, *P* = 0.9822 and 0.9848 respectively, Figures 1D,E). The time points for observation in the present study were selected based on previous studies and the preliminary study (Lubin et al., 2008; Dyrvig et al., 2015).

To determine the inherent effects of sevoflurane and to obviate the possible physiologic effects of anesthesia, C6 glioma cells were used. After exposure to 2.5% sevoflurane for 4 h, the mRNA levels of *Arc* (50.34 ± 5.635 vs. 100.00 ± 12.14, *P* < 0.0001), *Bdnf* (62.83 ± 12.92 vs. 100.00 ± 15.76, *P* = 0.0012), and *Reln* (73.77 ± 16.75 vs. 100.00 ± 20.99, *P* = 0.0378) in C6 glioma cells also decreased significantly (Figures 1F–H). The mRNA levels of *Egr1* (100.90 ± 12.53 vs. 100.00 ± 9.48, *P* = 0.8917) and *Ppp3ca* (104.10 ± 18.46 vs. 100.00 ± 19.79, *P* = 0.7185) were unchanged after anesthesia (Figures 1I,J). Taken together, these data suggested that anesthesia with sevoflurane decreased mRNA expression levels of *Arc*, *Bdnf* and *Reln* both *in vitro* and *in vivo* condition.

**FIGURE 2:**
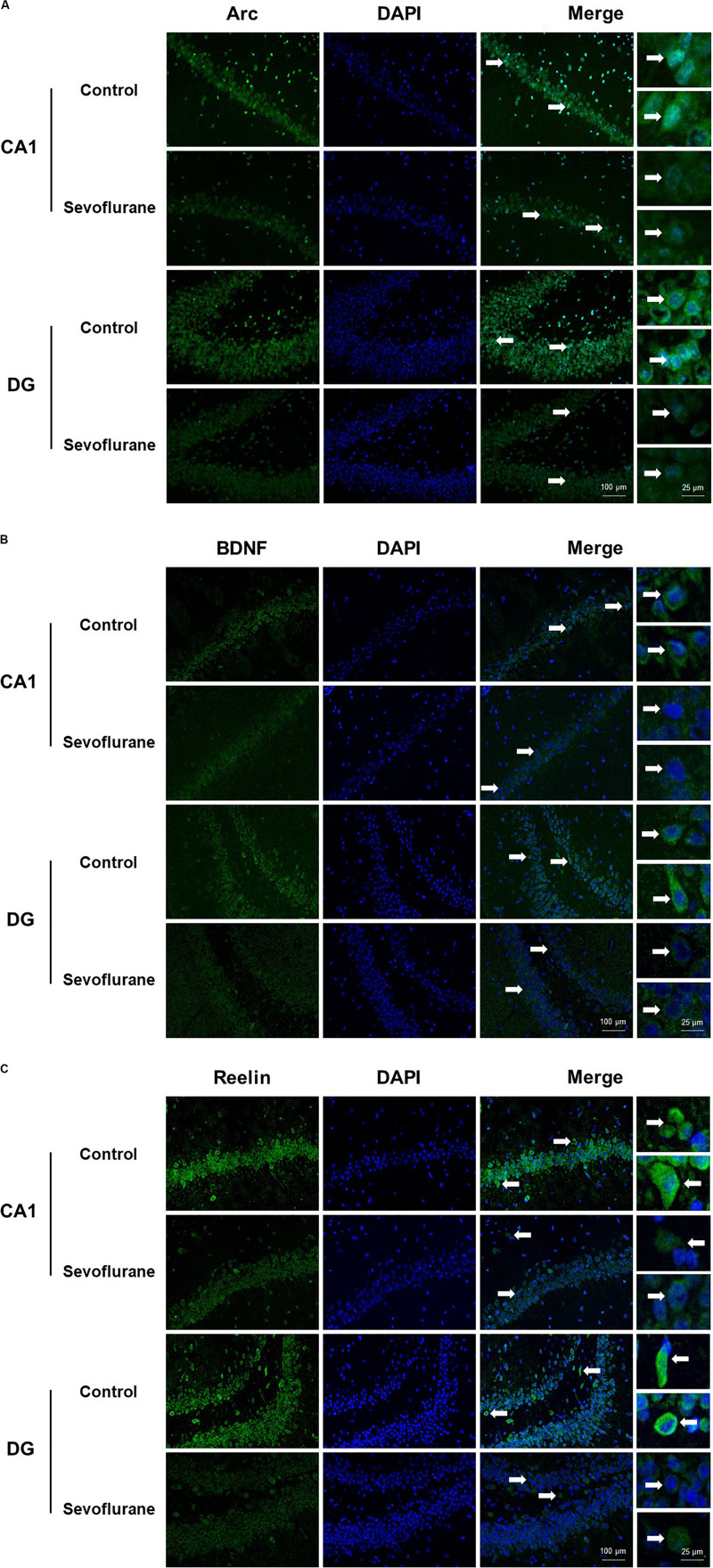
Immunoreactivities of Arc, BDNF, and Reelin decreased in the CA1 region and DG in the hippocampus of aged rats after sevoflurane anesthesia. Immunofluorescence images of hippocampal sections staining with antibodies (green), cell nuclei counter-stained with DAPI (blue) and merged images. Arc staining was detected in the cytoplasm and around the nucleus, and decreased in anesthesia group **(A)**. BDNF staining was most prominently observed in the cell bodies and axon terminals, and decreased in anesthesia group **(B)**. Compared to the anesthesia group, Reelin positive cells were more numerous and polymorphous in control condition **(C)**. In each panel, arrows point to the regions that present typical staining, which are provided as high magnification images in the corresponding right panels. Magnification 400×, scale bar 100 and 25 μm.

Next, we evaluated the immunolabeling of hippocampal Arc, BDNF and Reelin (encoded by *Arc*, *Bdnf*, and *Reln*, respectively) 3 h after exposure to after 2.5% sevoflurane for 4 h to determine whether the changes in protein production were corresponded to the alterations in gene transcription. Cytoplasmic and perinuclear Arc staining were detected in the pyramidal cells within CA1 region and in the granule cells within the dentate gyrus (DG). The staining decreased significantly in the sevoflurane group compared to the control condition (Figure 2A). BDNF staining was most prominently observed in the cell bodies and axon terminals in the pyramidal cells within CA1 region and in the granule cells within DG, and the staining decreased significantly in the sevoflurane group (Figure 2B). Reelin is a large secreted extracellular matrix glycoprotein and has been reported to accumulate in oligomeric amyloid-like plaques in the hippocampus of several aged species (Knuesel et al., 2009). Compared to the anesthesia group, Reelin positive cells in the control condition were more numerous and polymorphous, with smaller interneuron dots possibly representing extracellular Reelin aggregates (Figure 2C).

### Sevoflurane Anesthesia Induced Cognitive Impairment in Aged Rats

**FIGURE 3:**
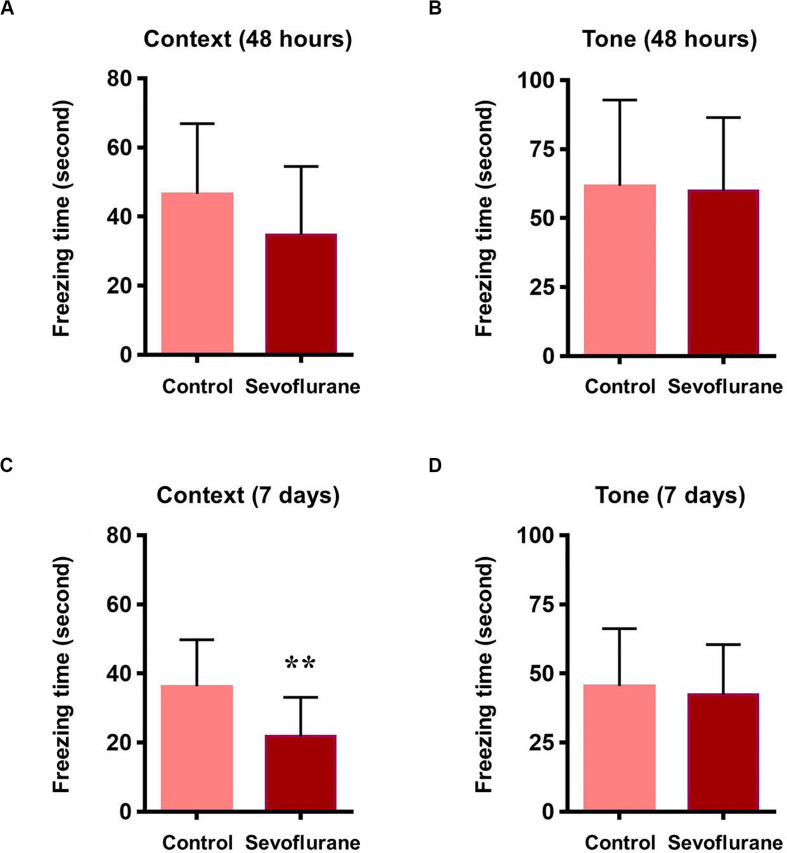
Sevoflurane induced hippocampus dependent memory impairment in aged rats 48 h after sevoflurane anesthesia, the freezing time did not change in both contextual and tone test of fear conditioning test **(A,B)**. 7 days after sevoflurane anesthesia, the freezing time reduced significantly in context test (hippocampus dependent, **C**), but did not change in tone test (hippocampus independent, **D**). *n* = 12 in each group. **P < 0.01 compared with the control group.

To assess whether sevoflurane anesthesia leads to hippocampus-dependent cognitive impairments, a subgroup of aged rats was subjected to the fear conditioning test (FCT) 48 h and 7 days after anesthesia. The results of the context test, mainly used to assess hippocampal function (Li et al., 2014), showed that sevoflurane anesthesia did not alter freezing time at 48 h (34.71 ± 19.77 vs. 46.59 ± 20.33, *P* = 0.1609, Figure 3A), however on day 7, rats exposed to sevoflurane displayed reduced freezing time (21.75 ± 11.32 vs. 36.29 ± 13.50, *P* = 0.0091, Figure 3C) compared to the control group, which suggested that sevoflurane induced hippocampal-dependent cognitive deficits persisted for a relatively long time period. During the tone test, which is related to amygdala function (Li et al., 2014), sevoflurane anesthesia did not alter freezing time at 48 h (61.7 ± 31.05 vs. 59.87 ± 26.55, *P* = 0.8777, Figure 3B) or on day 7 (45.49 ± 20.75 vs. 42.3 ± 18.19, *P* = 0.6933, Figure 3D), suggesting that amygdala function is not grossly impaired.

### Sevoflurane Altered *Dnmts* and *Mecp2* Expression in the Hippocampus of Aged Rats and C6 Glioma Cells

**FIGURE 4:**
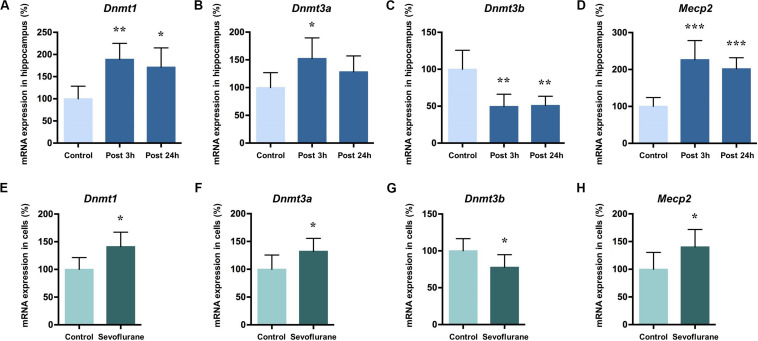
Sevoflurane increased *Dnmt1*, *Dnmt3a*, and *MeCP2* mRNA levels and decreased *Dnmt3b* mRNA levels in the hippocampus of aged rats and C6 glioma cells. The mRNA levels of *Dnmt1*, *Dnmt3a*, and *MeCP2* in the hippocampus of aged rats increased significantly 3 h afte*r* 4 h 2.5% sevoflurane anesthesia as compared with control condition, and the mRNA levels of *Dnmt1* and *MeCP2*, but not *Dnmt3a*, still increased significant 24 h after anesthesia **(A,B,D)**. *Dnmt3b* mRNA levels in the hippocampus of aged rats decreased significantly 3 h and 24 h after anesthesia **(C)**. The mRNA levels of *Dnmt1*, *Dnmt3a* and *MeCP2* in C6 glioma cells increased significantly after sevoflurane treatment **(E,F,H)**, and the mRNA levels of *Dnmt3b* in C6 glioma cells decreased significantly after treatment **(G)**. *n* = 6 in each group of *in vitro* and *in vivo* studies. *P < 0.05, **P < 0.01, and ***P < 0.001 compared with the control group.

DNA methylation is catalyzed by three DNA methyltransferases (DNMTs) in mammals, DNMT1, DNMT3a, and DNMT3b. DNMT3a and 3b catalyze *de novo* methylation, while DNMT1 is responsible for the maintenance of previously methylated sites in the adult brain (Robertson et al., 1999). Methyl-CpG binding protein 2 (MeCP2) typically acts as a transcriptional repressor that binds to methylated CpG and is involved in the maintenance of synaptic plasticity and cognitive functions in the mammal brain (Nan et al., 1997; Fasolino and Zhou, 2017). Given their roles in memory related neuronal plasticity in brain regions such as hippocampus (Lubin et al., 2008; Feng et al., 2010; Halder et al., 2016), we investigated whether *Dnmts* and *Mecp2* mRNA levels in the hippocampus of aged rats were altered by sevoflurane. The mRNA levels of *Dnmt1* (189.10 ± 35.94 vs. 100.00 ± 28.46, *P* = 0.0014), *Dnmt3a* (152.40 ± 37.15 vs. 100.00 ± 26.9, *P* = 0.0202) and *Mecp2* (226.70 ± 51.79 vs. 100.00 ± 24.20, *P* = 0.0001) significantly increased 3 h after sevoflurane exposure compared to the control. The increased transcriptional expression of *Dnmt1* (171.30 ± 43.52 vs. 100.00 ± 28.46, *P* = 0.0076) and *Mecp2* (201.80 ± 30.15 vs. 100.00 ± 24.20, *P* = 0.0005) persisted up to 24 h after sevoflurane treatment, but not for *Dnmt3a* (128.40 ± 28.70 vs. 100.00 ± 26.9, *P* = 0.2302, Figures 4A,B,D). However, the *Dnmt3b* mRNA levels decreased both 3 h and 24 h after sevoflurane exposure (49.51 ± 16.61 and 50.97 ± 12.47 vs. 100.00 ± 25.65, *P* = 0.0007 and 0.0009, respectively, Figure 4C).

The same pattern can be seen with *in vitro* experiment results. In C6 glioma cells, the mRNA levels of *Dnmt1* (141.00 ± 26.30 vs. 100.00 ± 21.41, *P* = 0.0142, Figure 4E), *Dnmt3a* (132.20 ± 23.42 vs. 100.00 ± 25.62, *P* = 0.0464, Figure 4F) and *Mecp2* (140.30 ± 31.41 vs. 100.00 ± 30.37, *P* = 0.0472, Figure 4G) increased and *Dnmt3b* mRNA levels decreased (100.00 ± 16.62, 77.72 ± 17.15, *P* = 0.0453, Figure 4H) after sevoflurane anesthesia. Assuming that *Dnmts* mRNA and protein levels correlate with DNA methylation levels (Fasolino and Zhou, 2017), therefore it is reasonable to speculate from these data that sevoflurane triggers hippocampal hypermethylation by dynamically upregulating *Dnmt1*, *Dnmt3a* and *Mecp2* expression.

### Sevoflurane Altered the Promoter Methylation Status of *Arc*, *Bdnf*, and *Reln* in the Hippocampus of Aged Rats and C6 Glioma Cells

**FIGURE 5:**
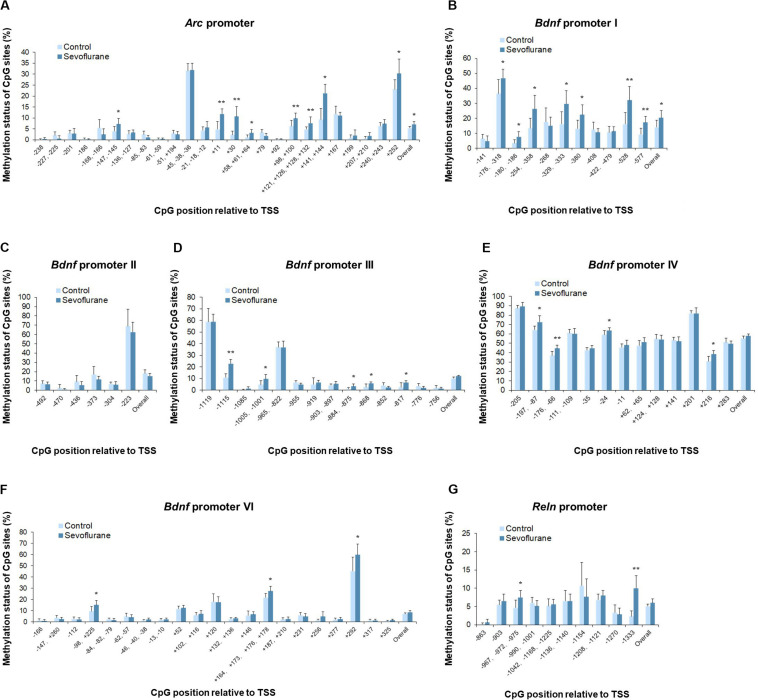
Sevoflurane induced hypermethylation status of *Arc*, *Bdnf* and *Reln* promoters in the hippocampus of aged rats. The frequency of methylation was observed at each CpG site in the *Arc*, *Bdnf* and *Reln* promoter regions in the hippocampus of aged rats after sevoflurane anesthesia. The overall methylation increased significantly in the *Arc* promoter and *Bdnf* promoter I, The DNA methylation increased at individual CpG sites in the *Arc* promoter, *Bdnf* promoter I, III, IV, VI, and *Reln* promoter, but not *Bdnf* promoter II **(A–G)**. *n* = 6 in each group. *P < 0.05 and **P < 0.01 compared with the control group.

To evaluate whether anesthesia induced hypermethylation in the hippocampus was responsible for the reduced transcripts, we then went on to investigate the methylation status of the promoter regions of *Arc*, *Bdnf* and *Reln* in the hippocampus of aged rats. Thus, methylation-specific qPCR was applied. Analysis of the overall methylation of the CpG sites revealed a substantial increase in the *Arc* promoter regions after anesthesia (*P* = 0.0143, Figure 5A). More specifically, a significant increase in DNA methylation was observed at the following individual CpG sites (*P* < 0.05): TSS (transcription start site) −147, TSS +11, TSS +30, TSS +58, TSS +98, TSS +121, TSS +141, TSS +207 and TSS +252. Also, sevoflurane induced a significant increase in the overall methylation at *Bdnf* promoter I (*P* = 0.0418, Figure 5B). The highly methylated CpG sites were: TSS −176, TSS −180, TSS −254, TSS −329, TSS −380, TSS −528, and TSS −577 (*P* < 0.05). The overall methylation of *Bdnf* promoter III, IV, VI was not different between control and sevoflurane groups (Figures 5D–F), but a significant increase in DNA methylation were observed in 5 CpG residues of *Bdnf* promoter III (TSS −1115, TSS −1005, TSS −884, TSS −868, and TSS −817; *P* < 0.05, Figure 5D), 4 residues of *Bdnf* promoter IV (TSS-197, TSS −176, TSS −24, and TSS +216; *P* < 0.05, Figure 5E) and 3 residues of *Bdnf* promoter VI (TSS −98, TSS +164, and TSS +292; *P* < 0.05, Figure 5F) in the sevoflurane group. *Bdnf* promoter II showed no change of methylation status in any of the CpG sites after anesthesia (Figure 5C). Overall methylation of *Reln* promoter was not different for control and anesthetized rats (Figure 5G). However, a significant increase in DNA methylation was observed in 2 CpG residues of *Reln* promoter (TSS −967 and TSS −1333) in the sevoflurane group (*P* < 0.05, Figure 5G).

**FIGURE 6:**
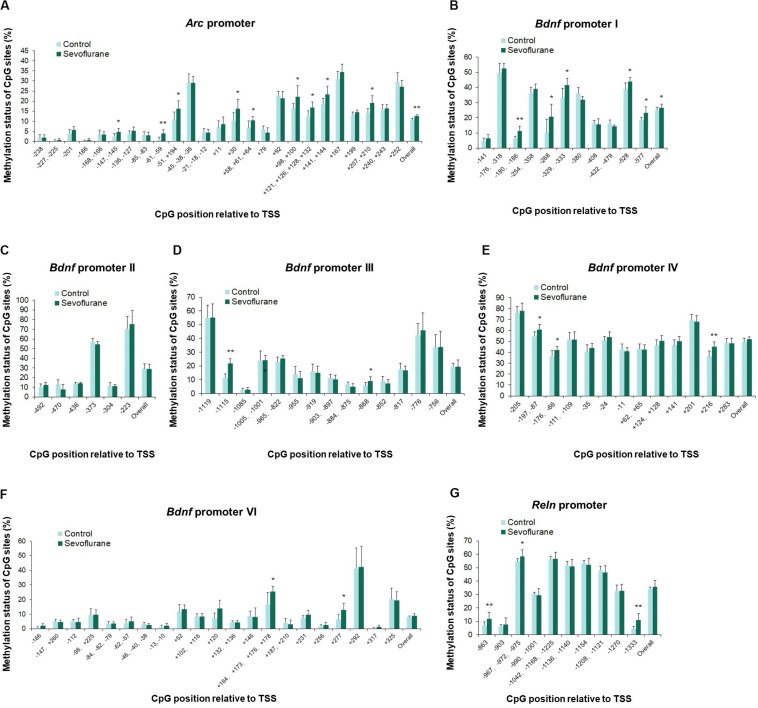
Sevoflurane induced hypermethylation status of *Arc* and *Bdnf* promoters in C6 glioma cells. The frequency of methylation was observed at each CpG site in the *Arc*, *Bdnf* and *Reln* promoter regions in C6 glioma cells after sevoflurane treatment. The overall methylation increased significantly in the *Arc* promoter and *Bdnf* promoter I, The DNA methylation increased at individual CpG sites in the *Arc* promoter, *Bdnf* promoter I, III, IV, VI and *Reln* promoter, but not *Bdnf* promoter II **(A–G)**. *n* = 6 in each group. *P < 0.05 and **P < 0.01 compared with the control group.

*In vitro* experiments with C6 glioma cells exhibited the same trends of increased methylation in the *Arc* promoter (*P* = 0.0037) and *Bdnf* promoter I (*P* = 0.0120) regions after sevoflurane treatment. *Arc* promoter was hypermethylated at TSS −147, TSS −61, TSS −51, TSS +30, TSS +58, TSS +98, TSS +121, TSS +141 and TSS +207 (*P* < 0.05, Figure 6A). And *Bdnf* promoter I was hypermethylated at TSS −180, TSS −268, TSS −329, TSS −528, and TSS −577 (*P* < 0.05, Figure 6B). We observed no significant difference in the overall methylation status of *Bdnf* promoter II, III, IV, VI and *Reln* promoters. However, sevoflurane induced several hypermethylated CpG sites in *Bdnf* promoter III (TSS-1115 and TSS −868; *P* < 0.05, Figure 6D), promoter IV (TSS-197, TSS −176, and TSS +216; *P* < 0.05, Figure 6E), promoter VI (TSS +120, TSS +164, and TSS +277; *P* < 0.05, Figure 6F), and *Reln* promoter (TSS −863, TSS −967, and TSS −1333; *P* < 0.05, Figure 6G). There was no change of methylation status in any of the CpG sites in *Bdnf* promoter II (Figure 6C). These data suggest that CpGs hypermethylation at particular sites in the promoter could involve in transcriptional suppression of *Arc*, *Bdnf* and *Reln* in response to sevoflurane treatment.

### DNA Methylation Manipulation Affected the Sevoflurane Induced Memory Gene Transcription Decrease in C6 Glioma Cells

**FIGURE 7:**
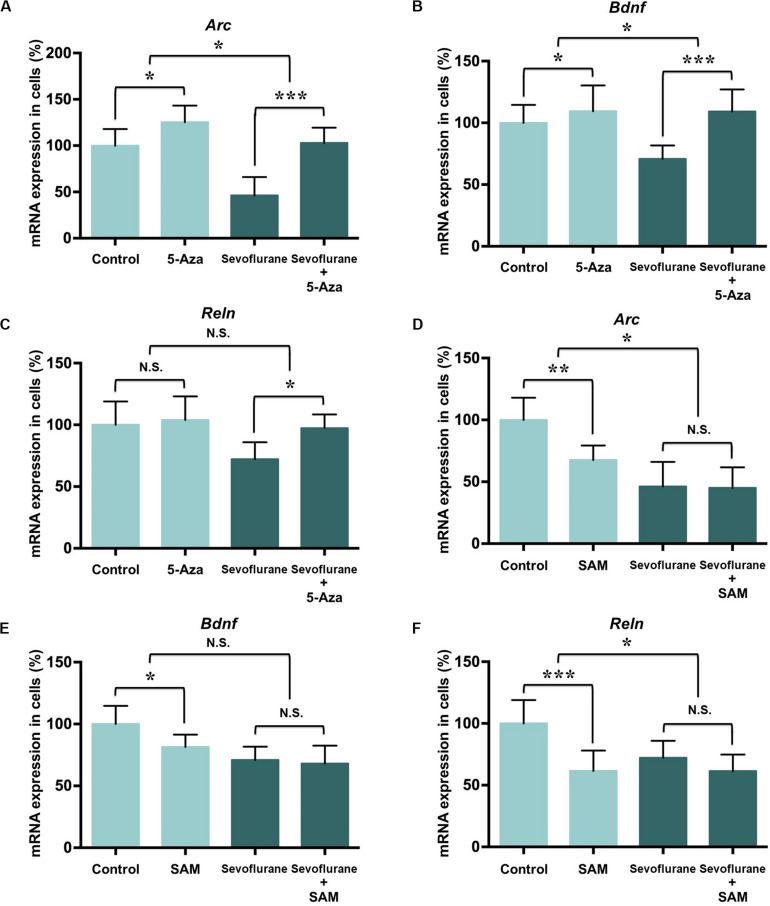
DNA methylation manipulation affected the sevoflurane induced *Arc, Bdnf* and *Reln* transcription decrease in C6 glioma cells. DNMT antagonist 5-Aza administration 60 min before sevoflurane treatment attenuated sevoflurane induced *Arc, Bdnf*, and *Reln* mRNA level decrease, but 5-Aza alone did not affect *Arc, Bdnf* and *Reln* mRNA levels in C6 glioma cells **(A–C)**. Methyl donor SAM C6 glioma cells administered with 60 min before treatment did not affect *Arc*, *Bdnf* and *Reln* mRNA level after sevoflurane treatment, but SAM alone decreased the mRNA expression of *Arc*, *Bdnf* and *Reln* mRNA levels in C6 glioma cells **(D–F)**. *n* = 6 in each group. *P < 0.05, **P < 0.01, and ***P < 0.001 compared with the control or sevoflurane group. N.S., not significant.

To determine whether the increased DNA methylation could account for decreased expression of *Arc*, *Bdnf* and *Reln* gene after sevoflurane, we firstly treated C6 glioma cells with pharmacological inhibitor 5-Aza-2′-deoxycytidine (5-Aza, 10 μM in cell culture) 60 min before sevoflurane anesthesia. 5-Aza is a nucleoside inhibitor incorporated into DNA by covalent binding and thereby blocking its DNA methyltransferase function (Navada et al., 2014). In the control condition, 5-Aza alone did not affect *Arc* (125.20 ± 18.01 vs. 100.00 ± 18.02, *P* = 0.0512), *Bdnf* (109.50 ± 20.87 vs. 100.00 ± 14.62, *P* = 0.5509) or *Reln* (103.90 ± 19.12 vs. 100.00 ± 18.92, *P* = 0.8985) mRNA levels. However, 5-Aza was found to attenuate decreased mRNA expression of *Arc* (46.17 ± 19.91 vs. 102.90 ± 16.61, *P* < 0.0001), *Bdnf* (70.86 ± 10.84 vs. 109.20 ± 17.87, *P* = 0.0013) and *Reln* (72.13 ± 13.79 vs. 97.27 ± 11.14, *P* = 0.0272) induced by sevoflurane. Two-way ANOVA yielded that the interaction of 5-Aza and sevoflurane treatment was significant for the mRNA levels of *Arc* (*P* = 0.0467), *Bdnf* (*P* = 0.0449), but not for *Reln* (*P* = 0.1217, Figures 7A–C).

Next, we treated C6 glioma cells with methyl donor S-Adenosyl methionine (SAM, 100 μM in cell culture) 60 min before sevoflurane treatment to induce DNA hypermethylation. Decreased the mRNA expressions of *Arc* (67.57 ± 11.69 vs. 100.00 ± 18.02, *P* = 0.0042), *Bdnf* (81.45 ± 9.97 vs. 100.00 ± 14.62, *P* = 0.0386) and *Reln* (61.57 ± 16.49 vs. 100.00 ± 18.92, *P* = 0.0009) were observed in the control condition. But SAM had no effect on *Arc* (45.11 ± 16.54 vs. 45.17 ± 19.91, *P* = 0.9222), *Bdnf* (68.11 ± 14.47 vs. 70.90 ± 10.85, *P* = 0.9136) and *Reln* (61.42 ± 13.40 vs. 72.13 ± 13.79, *P* = 0.4437) mRNA levels in the sevoflurane group. The interaction of SAM and sevoflurane treatment was significant for the mRNA levels of *Arc* (*P* = 0.0336) and *Reln* (*P* = 0.0441), but not for *Bdnf* (*P* = 0.1428, Figures 7D–F). Therefore, manipulating DNA methylation by 5-Aza is sufficient to restore suppressed memory genes transcription by sevoflurane.

### DNA Methylation Inhibition Attenuated the Sevoflurane Anesthesia Induced Hippocampal Memory Gene Transcription Decrease and Cognitive Impairment in the Aged Rats

**FIGURE 8:**
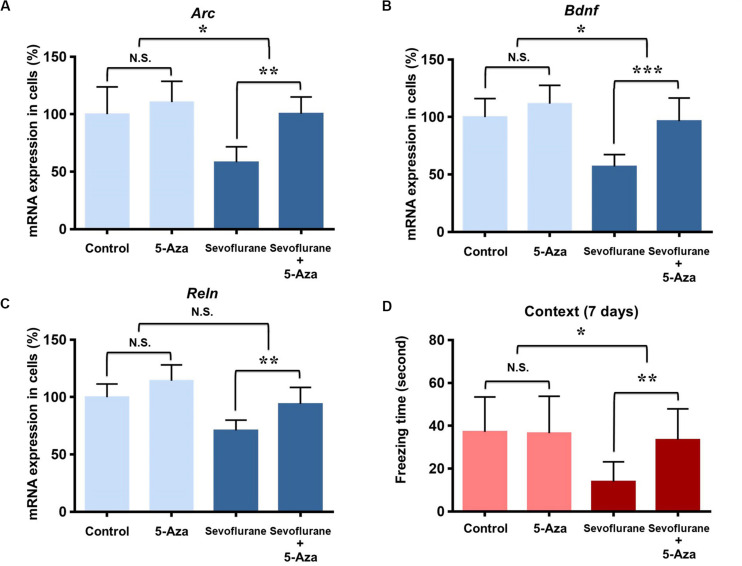
DNA methylation inhibition attenuated the sevoflurane induced hippocampal *Arc, Bdnf* and *Reln* transcription decrease and cognitive impairment in the aged rats. DNMT antagonist 5-Aza administration 30 min before sevoflurane anesthesia attenuated sevoflurane induced *Arc, Bdnf* and *Reln* mRNA level decrease, but 5-Aza alone did not affect *Arc, Bdnf* and *Reln* mRNA levels in the hippocampus of aged rats **(A–C)**. 5-Aza attenuated sevoflurane induced freezing time decrease, but 5-Aza alone did not affect the freezing time in the context test of FCT in aged rats at 7 days after anesthesia **(D)**. *n* = 6 in each group for qPCR, and *n* = 12 in each group for FCT. *P < 0.05, **P < 0.01, and ***P < 0.001 compared with the control or sevoflurane group. N.S., not significant.

As DNA methylation inhibition partially restored the down-regulation of memory gene transcription induced by sevoflurane *in vitro*, we further investigated its effects on hippocampal memory genes and cognitive function in the aged rats. 5-Aza treatment (0.5 mg/kg i.p., 30 min before anesthesia) attenuated decreased mRNA levels of *Arc* (100.5 ± 14.56 vs. 58.34 ± 13.35, *P* = 0.0012), *Bdnf* (96.49 ± 20.04 vs. 56.92 ± 10.41, *P* = 0.0007) and *Reln* (94.03 ± 14.35 vs. 70.94 ± 9.06, *P* = 0.0082) in the hippocampus resulting from sevoflurane anesthesia. But 5-Aza alone did not significantly affect hippocampal *Arc* (100.5 ± 14.56 vs. 58.34 ± 13.35, *P* = 0.5372), *Bdnf* (110.60 ± 18.12 vs. 100.00 ± 23.86, *P* = 0.3946) or *Reln* (114.30 ± 13.80 vs. 100.00 ± 11.43, *P* = 0.1127) mRNA levels. The interaction of 5-Aza and sevoflurane treatment was significant for the mRNA levels of *Arc* (*P* = 0.0435) and *Bdnf* (*P* = 0.0454), but not for *Reln* (*P* = 0.3947, Figures 8A–C).

Having confirmed that the cognitive impairment was evident at 7 days after sevoflurane in context test, and correlated with the changes in transcriptional and methylation profiles of memory genes in the aged rats, we then examined the effect of DNA methylation inhibition on cognitive function in context test at 7 days after anesthesia. The results indicated that 5-Aza treatment attenuated the reduced freezing time (33.47 ± 14.41 vs. 13.93 ± 9.268, *P* = 0.0042) in aged rats after sevoflurane anesthesia. But 5-Aza alone did not significantly affect the freezing time in the control condition (36.53 ± 17.24 vs. 37.17 ± 16.29, *P* = 0.9930). The interaction of 5-Aza and sevoflurane treatment was significant for a relatively long period (*P* = 0.0213, Figure 8D). Collectively, these findings indicate that decreased *Arc*, *Bdnf* and *Reln* expressions in the hippocampus are medicated by promoter hypermethylation, which is involved in the upstream mechanisms for sevoflurane induced cognitive impairment in aged rats.

## Discussion

In the present study, we found that sevoflurane anesthesia down-regulated the mRNA and protein levels of three memory genes, *Arc*, *Bdnf* and *Reln*, which were accompanied with promoter hypermethylation and increased *Dnmt1*, *Dnmt3a* and *Mecp2* expression, and finally impaired hippocampus dependent memory. Furthermore, inhibition of DNA hypermethylation by 5-Aza rescued sevoflurane induced memory gene expression decrease and cognitive impairment. These findings suggest that anesthesia induced epigenetic modulations could be responsible for the long term cognitive impairment in aged rats.

Cognitive processes, such as the fear conditioning test, require transcription of a wide array of genes encoding neurotrophins, pre-synaptic, and post-synaptic proteins and cytoskeletal elements responsible for different types of plasticity events and memory processes in hippocampus, a brain region vulnerable to the aging process (Lubin et al., 2008; Carmichael and Henley, 2018). Both loss and gain of function manipulations to these activity-regulated genes, including *Arc*, *Bdnf*, *Reln*, *Egr1* and *Ppp3ca*, have been associated with cognitive deficits during physiological aging and pathological conditions like dementia, neurodegenerative diseases and neuropsychiatric problems (Nonaka et al., 2014). In the present study, we report that attenuated transcriptions and protein synthesis of *Arc*, *Bdnf* and *Reln* genes may contribute to hippocampus dependent memory deficits in the aged rats after sevoflurane anesthesia. Combined with previous studies with isoflurane (Bunting et al., 2015; Dalla Massara et al., 2016), we further investigate the transcriptional regulatory mechanisms to drive the coordinated response of multiple memory genes to inhaled anesthesia.

Epigenetic changes in the brain are critical for the long term memory storage through altering genes transcription that are associated with synaptic plasticity during development and throughout adult life (Hwang et al., 2017). Growing number of studies suggest that anesthesia induces a variety of epigenetic modifications in neonatal brain, leading to developmental neurological disorders in animal models (Dalla Massara et al., 2016; Ju et al., 2016; Wu et al., 2016; Jia et al., 2017; Joksimovic et al., 2018). In the present study, we focused on the contribution of DNA methylation to memory genes during anesthesia in aged rats. DNMT inhibition can impair memory formation as well as long-term potentiation (LTP) at CA1 synapses (Feng et al., 2010). DNMT activity and global DNA methylation are decreased in rat brain during aging (Liu et al., 2009) and brain samples from AD patients (Liu et al., 2011). Our results showed that sevoflurane increased *Dnmt1*, *Dnmt3a* and *Mecp2* expressions, resulted in DNA hypermethylation in the promoter regions of *Arc*, *Reln* and *Bdnf*, and suppressed transcription of these genes. These results are consistent with work in the hippocampus by Levenson et al. (2006), Dyrvig et al. (2015).

DNA methylation has been reported to play key roles in memory formation and maintenance. Accumulating evidence suggests that DNA methylation is dynamically regulates the function of neurons in response to learning experience (Miller et al., 2010). Abnormal hypermethylation or hypomethylation could both lead to memory dysfunction (Ehrlich, 2019), and correlate with multiple neurological disorders, including AD (manifested as neuronal loss and dementia) (Semick et al., 2019). DNA methylation modulation related gene transcription variations play a key role during these processes. DNA methylation occurs preferentially on CpG sites. There are a large number of CpG islands in promoter regions, which could change the original configuration of the promoters in the genes, interfere the combination of specific transcription factors and specific recognition sites, affect the transcription of downstream genes, and result in the abnormal transcription of memory genes (Zhu et al., 2016).

Our intervention studies indicated that SAM decreased the expressions of *Arc*, *Bndf* and *Reln* in control condition (the DNA methylation status were in normal levels and relatively sensitive to the supplementation of methyl donor), but not after sevoflurane in cells (with anesthetic induced hypermethylation). Meanwhile, 5-Aza affect the gene expressions after sevoflurane exposure (with hypermethylation), but not in control condition. The interactions were significant in both studies, which indicated that DNA methylation modification could be a pivotal mechanism for the effects of sevoflurane exposure on memory genes. Furthermore, accompanied with memory gene expression increase, 5-Aza also attenuated sevoflurane induced cognitive impairment in aged rats. Thus, the combination of external factor (sevoflurane) and internal factor (aging) increased the susceptibility to DNA methylation in the hippocampus, which in turn led to the decrease of memory gene expressions and cognitive impairment.

Among these memory genes, BDNF is a member of neurotrophins, which influence neuronal proliferation and differentiation, modulate LTP induction and maintenance, and contribute to the maintenance of synaptic plasticity in the central neurons (Lubin et al., 2008). The rodent *Bdnf* gene has nine exons, eight of which have their own promoter contains multiple promoters that are specifically regulated by different stimuli (Aid et al., 2007). Kainic acid, a glutamate analog, induced transcription of *Bdnf* exons I, IV, V VII, VIII and IXA in rat hippocampus while N-methyl-d-aspartate (NMDA) treatment identified *Bdnf* exons II and IV, but not I and III, as fast reacting exons (Aid et al., 2007; Palomer et al., 2016a). This epigenetic modification of *Bdnf* transcription may largely change its expression and contribute to the pathogenesis of several neurological disorders. For instance, aging reduced basal levels, while fear conditioning increased expression of total *Bdnf* mRNA and exon IV specific transcripts (Chapman et al., 2012). In animal models of AD, occupancy of HDAC2 in the promoter region of *Bdnf* exon IV contribute to the reduction of BDNF in APP/PS1 mice (Hsiao et al., 2017) and infusion of amyloid fibrils into the hippocampus of rats induces HDAC2 occupancy at promoter VI of *Bdnf* and thus decreases *Bdnf* expression (Hendrickx et al., 2014). In our study, increased cytosine methylations were observed in the promoter region of *Bdnf* exon I, III, IV, and VI, which may contribute to the long lasting hippocampal BDNF reduction induced by general anesthesia.

The immediate early gene *Arc*, which interacts with the NMDA receptor complex, is activated during synaptic activation and memory consolidation (Gao et al., 2018). *Arc* promoter contains CpG sites and intragenic locus that recruit methyl-DNA binding proteins (Epstein and Finkbeiner, 2018). Penner et al. (2011) reported that increased methylation of *Arc* in aged rats might be responsible for age related processes. Calcium influx through NMDA receptors could activate a signaling cascade resulting in CREB phosphorylation and CREB binding protein association. Subsequently, phosphorylated MeCP2 is dissociated from methylated DNA and leads to transcriptionally active chromatin, then affects *Arc* (Epstein and Finkbeiner, 2018) and *Bdnf* (Palomer et al., 2016a, b) expression. Inhaled anesthetic at clinically relevant concentrations has been shown to inhibit NMDA currents (Haseneder et al., 2013). Thus, NMDA receptor activation could be the upstream mechanism for the epigenetic regulation of *Arc*, *Bdnf* and other memory genes.

*Reln* encodes an extracellular matrix protein that contacts postsynaptic dendritic spines to controls glutamatergic neurotransmission through differential modulation of NMDA and AMPA receptor activity, and is critical for synaptic plasticity and memory formation (Doehner and Knuesel, 2010; Telese et al., 2015). Impaired Reelin signaling has a devastating effect on the gross morphology of the hippocampus and involves in pathological forms of aging, such as late-onset AD (Doehner and Knuesel, 2010; Pujadas et al., 2014). Consistent with the present results, changes in MeCP2 binding and hypermethylation in *Reln* promoter are associated with major mental illnesses such as Schizophrenia, bipolar disorder (Mitchell et al., 2005; Zhubi et al., 2014; Teroganova et al., 2016). The results showed that 5-Aza, DNA methyltransferase inhibitor, has a potential therapeutic effect on anesthesia induced memory impairment through affecting the methylation status of *Arc*, *Bdnf* and *Reln*. Moreover, 5-Aza has also been reported to induce demethylation by blocking DNMT enzyme activity at *Arc* promoter in rat hippocampus (Singh et al., 2015), *Bdnf* promoter I in mouse Neuro-2a cells (Ishimaru et al., 2010), *Reln* promoter in NT-2 neuronal precursor cells (Kundakovic et al., 2009), and restore recognition memory consolidation in ovariectomized mice (Zhao et al., 2010).

There are several limitations in the present study. First, there are multiple mechanisms during the sevoflurane related cognitive impairment, including neuroinflammation, metabolic alterations, electrophysiological changes, etc. DNA methylation variation and gene expressions could involve in these processes, and related investigations, including electrophysiology, should be performed in the future investigations. Second, as behavioral abnormal has multiple manifestations, combined behavior tests with Morris water maze, FCT and open field test should be performed in the future investigation to provide a comprehensive behavioral function during anesthesia and hippocampal DNA methylation modulation.

Taken together, sevoflurane anesthesia significantly reduced the expressions of *Arc*, *Bdnf* and *Reln* through inducing promoter hypermethylation in the hippocampus, which substantially contributed to cognitive impairment in aged rats. These impairments could be attenuated by 5-Aza pretreatment. Our study provides an epigenetic understanding for the pathophysiology of cognitive impairment induced by general anesthesia in the aged brain.

## Data Availability Statement

The datasets presented in this study can be found in online repositories. The names of the repository/repositories and accession number(s) can be found in the Dryad Digital Repository (https://doi.org/10.5061/dryad.69p8cz8xw).

## Ethics Statement

The animal study was reviewed and approved by the Peking University Biomedical Ethics Committee Experiment Animal Ethics Branch.

## Author Contributions

CN designed the project, performed the experiments, analyzed the data, and wrote and revised the manuscript. MQ wrote the original draft of the manuscript. JG, YT, and SL contributed to data analysis and manuscript revision. YQ and NY contributed to the experiments. HZ designed and supervised the project, and revised the manuscript. All authors read and approved the final manuscript.

## Conflict of Interest

The authors declare that the research was conducted in the absence of any commercial or financial relationships that could be construed as a potential conflict of interest.

## Footnotes

1 https://doi.org/10.5061/dryad.69p8cz8xw

2 http://www.epidesigner.com/index.html
